# The effect of intradialytic exercise on dialysis patient survival: a randomized controlled trial

**DOI:** 10.1186/s12882-023-03158-6

**Published:** 2023-04-17

**Authors:** Mohammad Ali Tabibi, Bobby Cheema, Nasrin Salimian, Hugo de Luca Corrêa, Saghar Ahmadi

**Affiliations:** 1 Department of Exercise Physiology, Pardis Specialized Wellness Institute, Isfahan, Iran; 2 School of Health Sciences, Western Sydney University, Campbelltow, NSW 2560 Australia; 3 National Institute of Complementary Medicine Health Research Institute, Western Sydney University, Westmead, NSW 2145 Australia; 4 Translational Health Research Institute, Western Sydney University, Campbelltown, NSW 2560 Australia; 5 Department of Research and Development, Pardis Specialized Wellness Institute, Isfahan, Iran; 6 Graduate Program of Physical Education, Catholic University of Brasilia, Distrito Federal, Brazil; 7 Department of Health and Palliative Care, Pardis Specialized Wellness Institute, Isfahan, Iran

**Keywords:** Mortality, Exercise during dialysis, Physical function, Nutritional status, Hematological Parameters, Survival

## Abstract

**Background:**

Patients with kidney failure have a high mortality rate. This study aimed to evaluate the effect of intradialytic exercise on survival in patients receiving hemodialysis (HD)**.**

**Methods:**

In this randomized controlled trial conducted in a HD center in Iran, adult patients receiving chronic HD were randomized to intradialytic exercise (60 min) in the second hour of thrice weekly dialysis for 6 months (intervention) or no intradialytic exercise (control). The primary outcome was survival rate at 12 months. Secondary outcomes were serum albumin, hemoglobin, hematocrit, red blood cell count, serum calcium, serum phosphorous, parathyroid hormone, physical function (6-min walk test) and nutritional status (Geriatric Nutritional Risk Index) during the first 6 months. The trial follow-up period was 12 months.

**Results:**

The study included 74 participants (44 males) with an age average of 64 ± 12 years old and a dialysis history of 27 ± 12 months, randomized to intervention (*n* = 37) or control (*n* = 37). Compared with controls, 1-year survival was higher in the intervention group (94% vs 73%, *P* = 0.01). The hazard ratio in univariate analysis in intervention group was 0.17 (95% CI 0.04–0.8; *P* = 0.02) compared to that in control group. During the 6-month intervention period, significant between-group changes were observed in all secondary outcomes between the intervention and control groups.

**Conclusion:**

Intradialytic exercise performed for at least 60 min during thrice weekly dialysis sessions improves survival in adult patients receiving HD.

**Trial registration:**

ClinicalTrials.gov Identifier: NCT04898608. Retrospectively registered on 24/05/2021. Registered trial name: The Effect of Intradialytic Exercise on Dialysis Patients Survival.

**Supplementary Information:**

The online version contains supplementary material available at 10.1186/s12882-023-03158-6.

## Background

Chronic kidney disease (CKD) is a growing, global public health problem leading to an exponential increase in the number of patients experiencing kidney failure requiring treatment with life-saving kidney replacement therapy (KRT), including hemodialysis (HD), peritoneal dialysis (PD) or kidney transplantation [1]. HD is the major treatment modality worldwide, accounting for 69% of all KRT and 89% of all dialysis, and is associated with high rates of functional impairment, morbidity, hospitalization, and mortality (10–30 times higher than people with normal kidney function) [2–5]. These outcomes are an urgent priority for patients, caregivers and healthcare professionals [6, 7].

A significant factor underpinning these poor outcomes in HD patients is low physical activity related to high rates of comorbidities (such as cardiovascular disease), protein energy wasting, sarcopenia, decreased physical function, decreased aerobic capacity, enforced inactivity during thrice weekly HD sessions, and post-dialysis fatigue [3, 8]. Indeed, the activity rates of patients receiving HD is between 20–50% that of healthy people [9]. Prospective observational studies of patients receiving HD have reported a dose-dependent association between physical activity and both all-cause mortality and cardiovascular mortality [9, 10]. Increasing physical activity through regular exercise may therefore be an important strategy for improving outcomes in patients receiving HD. Exercise training, particularly a combination of resistance and aerobic exercises, has been reported to improve a number of parameters in HD patients, including physical function, dialysis small solute clearance, mood, appetite, nutrient intake and quality of life [11–13].

A systematic review and meta-analysis of 20 randomized controlled trials involving 677 participants receiving HD demonstrated that exercise training increased exercise capacity (peak VO_2_), walking capacity (6-min walk test) and both the physical and mental component scores of health-related quality of life (SF-36) [11]. However, the certainty of evidence was substantially reduced by high risks of bias, imprecision and inconsistency (heterogeneity). Moreover, the effects of exercise on mortality in HD patients remain uncertain.

The aim of this study was to evaluate the effects of intradialytic exercise on survival in patients receiving HD. A secondary aim of the study was to assess the effect of intradialytic exercise on clinical outcomes associated with patient survival.

## Methods

### Trial design

This study is an open-label, parallel arm, randomized controlled trial with blinded end-points, which was conducted in a medical center in Iran. Recruitment occurred between January 25, 2020 and 2 February 2020. Follow-up continued until August 5, 2021.

### Participants

Individuals were eligible to participate in the study after meeting all of the following inclusion criteria: 1) age ≥ 18 years; 2) receiving regular HD 3 times a week; 3) on HD for at least 1 year, 4) absence of a history of myocardial infarction within the past 3 months; 5) permission from their physician to participate; and, 6) had capacity to provide informed consent to participate in the study. Individuals were excluded if they met any of the following exclusion criteria: 1) cardiac instability (angina, decompensated congestive heart failure, severe arteriovenous stenosis, uncontrolled arrhythmias, etc.); 2) active infection or acute medical illness; 3) hemodynamic instability (systolic blood pressure < 90 mmHg or mean arterial pressure < 60 mmHg); 4) labile glycemic control (extreme swings in blood glucose levels, causing hyperglycemia or hypoglycemia); 5) inability to exercise (e.g. lower extremity amputation with no prosthesis); 6) severe musculoskeletal pain at rest or with minimal activity; 7) inability to sit, stand or walk unassisted (walking device such as cane or walker allowed); or 8) shortness of breath at rest or with activities of daily living (NYHA Class IV).

### Trial procedures

Before starting the study, some educational and motivational posters were installed in the dialysis center, so that all patients became familiar with the benefits of exercise and especially intradialytic exercise. Then principal investigator described the side-effects of inactivity and sedentary lifestyle to all patients. Actually, he encouraged all the patients to be active and gave them awareness about the intradialytic exercise and how it can help them.After providing written informed consent, eligible patients received a baseline assessment. Data were collected on demographic characteristics (age, sex and time on hemodialysis), primary cause of kidney failure, and comorbidities (atherosclerotic heart disease, congestive heart failure, cerebrovascular accident/transient ischemic attack, peripheral vascular disease, dysrhythmia, and other cardiac diseases, chronic obstructive pulmonary disease, gastrointestinal bleeding, liver disease, cancer, and diabetes). Comorbidities were quantified using Charlson comorbidity index (CCI) established for dialysis patients, which included the underlying cause of kidney failure, as well as 11 comorbidities [14].

Participants were then randomized in a 1:1 ratio to either the intervention group or control group. The randomization sequence was generated by a study biostatistician who was not otherwise involved in the study using a computer-generated random schedule (using Stata 16, Stata Crop, College Station, Tx). Allocation concealment was safeguarded through the use of sequentially numbered, sealed, opaque envelopes by a specified staff member who was not involved in the study.

### Intervention

Subjects in the intervention group performed concurrent intradialytic exercise during the 2nd hour of dialysis (60-min exercise sessions three times a week) for 6 months. The intervention was a combination of aerobic and resistance exercises. Workout time at the beginning was 30 min and gradually increased to 60 min. Each workout session included 5 min of warm-up, aerobic exercises, resistance exercises and finally 10 min of stretching exercises to cool down. The fistula arm was kept stationary thoroughly the exercise session, with necessary precautions taken into consideration. Also, the exercise protocol was not performed on the arm with AV fistula.

Exercises were individualized in a way that matched the level of physical fitness of participants (See Aditional File 1). Aerobic exercises consisted of continuously performed specified movements, such as moving legs back and forth, shoulder abduction and adduction (hand without fistula), flexing and extending the knee, internally and externally rotating the leg, and abducting and adducting the leg, in time with a played beat**.**

The rhythm of continuous movements was adjusted by the beats per minute of the music. This meant that participants had to coordinate the movements of their arms and legs with the beats per minute of the song being played to them. In this way, the speed and intensity of aerobic exercise was controlled by the rhythm. Resistance training was performed in a semi-recumbent position and included exercises for the upper and lower limbs as well as core strength exercises using body weight, weight cuffs, dumbbells, and elastic bands of varying intensity. Chest press, shoulder press, triceps extension, straight arm shoulder flexion, shoulder horizontal abduction,seated row, supine grip, prone grip, neutral grip, bicep curl, leg abduction, plantarflexion, dorsiflexion, straight-leg/bent knee raises, knee extension, and knee flexion were all part of the resistance training program.

All of the exercises were prescribed by an exercise physiologist. He accompanied patients to monitor the performance of the exercises. If the patient had a problem in performing the exercises, he helped them and answered any questions raised. At the end of each session, the exercise physiologist reviewed the adherence checklists. If a person had not attended an exercise session, a counseling session were held with the presence of a nephrologist and the exercise physiologist. The reason for the individual's non-participation was investigated and the positive effects of exercise were explained through motivational statements. In addition, if possible, the positive effects of exercise that have had happened to them so far were explained as an incentive for them to participate more in training sessions.

Participants in the control group did not undertake any specific physical activity during dialysis. All participants were followed for 12 months.

All other pharmacological, dialysis, dietary and management protocols were identical for participants in both groups. (Despite of differences between some hematological parameters at baseline, different medications were not used for intervention group.) All participants received normal bicarbonate hemodialysis, which was carried out three times a week for an average of 4 h. Volumetric ultrafiltration control was available on all machines. The standard dialysate flow rate was 500 mL/min and blood flow rates were prescribed according to the participant’s needs. Automated methods were applied to perform dialyzer reuse uniformly.

### Blood sampling

Baseline blood samples were collected one day before the start of the exercise session. Exercise began at the mid-week dialysis session. After the end of the 36th session (end of the third month) and after the end of the 72nd session (end of the sixth month), subsequent blood samples were collected the day before the midweek dialysis session. The control group was assessed at the same time points. On a nondialysis day, blood samples were taken from the arterial needle after at least an 8-h fast. Approximately 30 ml of blood were collected and centrifuged for 15 min at 20 °C and 2500 g. Plasma was next pipetted into cryotubes and stored at -80 °C in a freezer that was electronically monitored. All samples were measured in duplicate, in line with the manufacturer's suggested protocol, and within the manufacturer's specified range of acceptable variation and sensitivity.

### Outcomes

The primary outcome measure was 1-year survival. Time frame started by ending the intervention (6th month). Information about the time and cause of death were extracted from participants’ medical records. This included information recorded in the CMS-2746 form.

Secondary outcome measures included changes in serum albumin (g/dL), hemoglobin (g/dL), hematocrit (%), red blood cell count (× 10^6^/µL), serum calcium (mg/dL), serum phosphorous (mEq/L), and parathyroid hormone (PTH) (pg/mL) over time. Other secondary outcomes were physical function and nutritional status. These outcomes were evaluated at baseline, 3 months and 6 months.

Physical function was evaluated with the 6-min walk test (6MWT), which is a functional examination of exercise capacity and was performed according to the American Thoracic Society guidelines [15]. In brief, the test was performed indoors on a 30 m straight course. Participants were instructed to walk as fast as possible for 6 min. Walking aids were allowed and recorded. At the end of the 6-min period, the distance was measured.

Nutritional status was assessed by Geriatric Nutritional Risk Index (GNRI), as reported by Bouillanne et al. [16] and modified for older patients, as reported by Yamada et al. [17]. The index was calculated as follows: GNRI = (14.89 albumin (g/dlL) + (41.7 x [body weight/ideal body weight]). When a participant's actual weight was greater than their ideal weight, the body weight/ideal body weight ratio was set to 1 by default. Instead of utilizing the Lorentz formula from the original GNRI equation to determine the ideal body weight, the value obtained from the participant's height and a body mass index of 22 was used [16]. Lower GNRI is a significant predictor of bone mineral disorders, cardiovascular events and all-cause mortality in dialysis patients [18–20].

Safety outcomes included all serious adverse events and adverse events.

### Adherence

Intervention adherence was defined as the number of sessions performed divided by the number of sessions offered, multiplied by 100.

### Blinding

Due to the nature of the intervention, it was not feasible to blind participants or study staff.

However, outcome assessors and data analysts were blinded to participants’ treatment allocations.

### Sample size

The sample size was calculated by NCSS PASS 16.0 software. The model was established according to the log-rank test of survival analysis (bilateral side), with α = 0.05 and power 1 – β = 0.8. Due to the fact that there had been no prior randomized controlled trials evaluating the effect of exercise on survival in dialysis patients, the results of a non-randomized study of the association of changes in physical activity with survival in dialysis patients were used to inform sample size calculations [21]. Assuming a hazard ratio of 0.25 between the intervention and control groups and a drop-out rate of 20%, 74 participants (37 per group) were required to provide 80% power.

### Statistical analysis

Data are presented as frequency (percentage), mean ± standard deviation, or median and interquartile range, depending on data type and distribution. A detailed statistical analysis plan was prepared and completed prior to database lock. The primary outcome of survival was analyzed by Kaplan–Meier analysis and log rank test. Overall survival was calculated from the date of the end of intervention to the date of death from any cause. Participants remaining alive were censored at the date of last follow-up. Cox regression model was constructed to evaluate the effect of intervention on survival rate. Secondary outcomes were evaluated using Repeated Measure ANOVA and Friedman test. Statistical analyses were performed using IBM SPSS software 25. *P* values less than 0.05 were considered statistically significant.

## Results

**Fig. 1:**
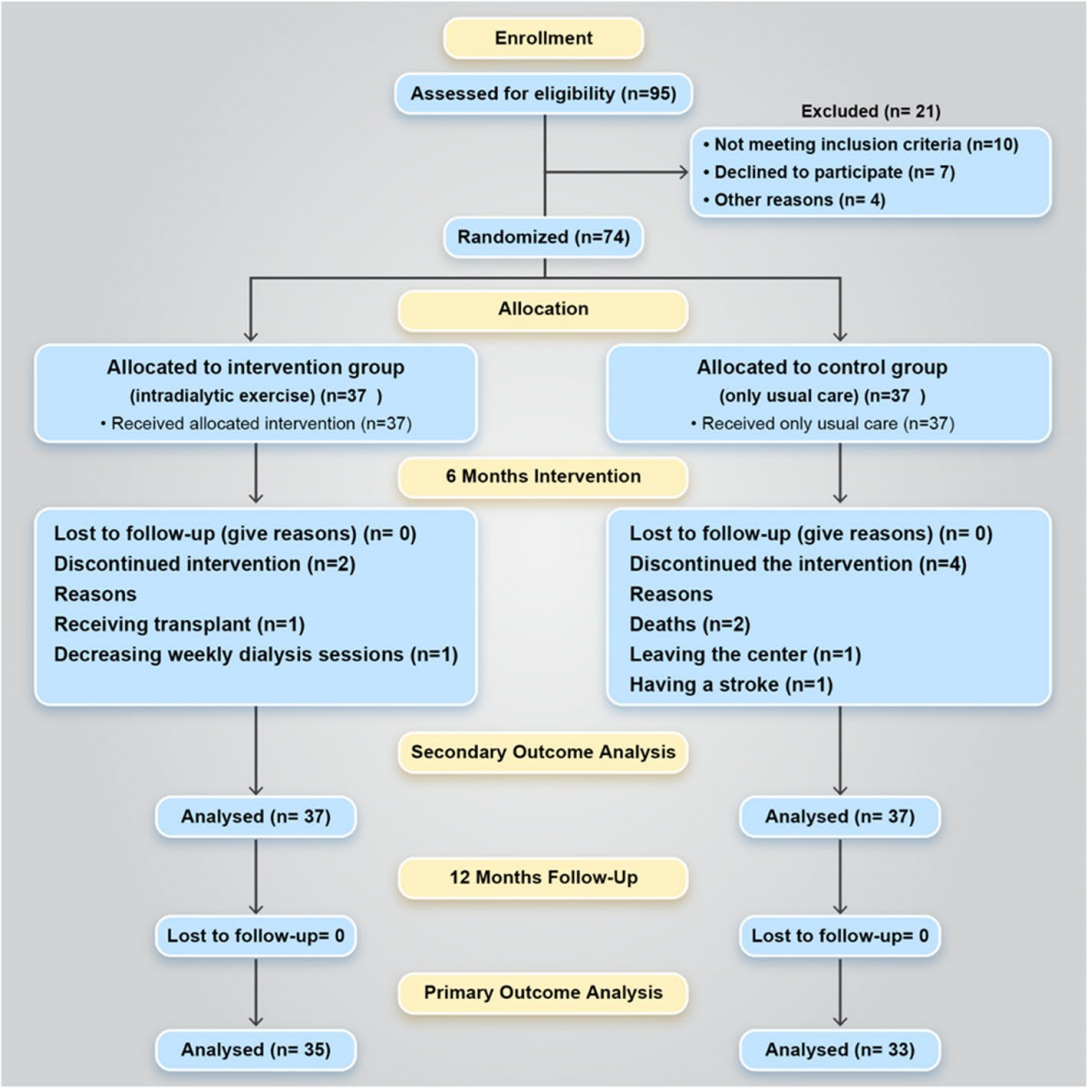
Participant flow during the study

Overall, 95 patients were assessed for eligibility, of whom 74 were consented and randomized. The corresponding flowchart is presented in Fig. 1

During the 6-month intervention period, 2 participants in the intervention group and 4 participants in the control group were excluded from the intervention. Every patient who was lost to follow-up (for any reason except death) during 12-month follow-up was censored.

### Baseline characteristics

Baseline characteristics were balanced between the assigned treatment groups (Table 1).

**Table1:** Baseline characteristics of patients

	Intervention (*n* = 37)	Usual Care (*n* = 37)
Sex Male	21 (57%)	23 (62%)
Age (year)	62 ± 13	65 ± 11
Hemodialysis history (months)	29 ± 10	26 ± 9
Primary kidney disease		
Diabetes	16 (43%)	20 (54%)
Hypertension	10 (27%)	11 (30%)
Glomerulonephritis	5 (14%)	3 (8%)
Other	6 (16%)	3 (8%)
CCI	6 ± 2	7 ± 2

Values are as mean ± (standard deviation) or n (%)

CCI Charlson Comorbidity Index

The adherence rate was 81% ± 6%.

### Primary outcome

Before the end of initial 6-month period during which the exercise intervention was being performed, no one in the intervention group and two participants in the control group died.

**Fig. 2:**
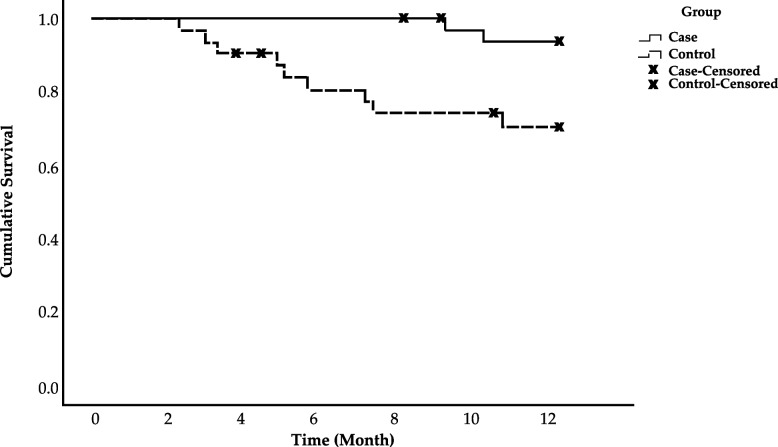
Kaplan–Meier survival curve for patients randomly allocated to intradialytic exercise for 6 months (intervention) or usual care (control). The difference between the groups was statistically significant (*p* = 0.01)

Two participants (6%) in the intervention group died during the follow-up period due to cardiovascular disease (*n* = 1) and unknown cause (*n* = 1). In contrast, 9 participants (27%) in the control group died due to cardiovascular disease (*n* = 3), cerebrovascular disease (*n* = 2), infection (*n* = 1), other causes (*n* = 2) and unknown cause (*n* = 1). The cumulative survival rate in the control group was significantly lower than that in the intervention group (Log rank statistics = 6.5, *P* = 0.01) (Fig. 2). Similar results were found in univariable Cox regression model analyses (Table 2).

**Table 2:** Cox regression model for all-cause mortality

Parameter	Univariate analysis		
	Hazard ratio	(95% CI)	*P* value
Intervention group	0.17	(0.04–0.8)	0.02*

^*^p < 0.05 significant

The hazard ratio is an estimate of the ratio of how often mortality happens in the intervention group compared to how often it happens in the control group. A confidence interval for hazard ratio, straddling 1.0 would reflect a statistically non-significant result

### Secondary outcomes

Significant between-group changes during the 6-month intervention period were observed in all secondary outcomes (Table 3) between the intervention and control groups. Specifically, serum albumin, hemoglobin, red blood cell count, serum calcium, physical function (6MWT) and nutritional status (GNRI) tended to increase in the intervention group, but remained relatively stable in the control group. In contrast, serum parathyroid hormone and phosphorus levels significantly decreased in the intervention group but remained relatively stable in the control group (Table 3).

**Table 3:** Change of secondary outcomes and the results of longitudinal analysis

	Intervention			Usual care				
	Baseline (*n* = 37)	3rd months (*n* = 36)	6th months (*n *= 35)	Baseline (*n* = 37)	3rd months (*n* = 35)	6th months (*n* = 33)	Partial Eta Square	Group× Time
Alb (g/dl)	3.5 ± 0.3	3.7 ± 0.3	4 ± 0.3	3.7 ± 0.3	3.6 ± 0.3	3.5 ± 0.2	0.5	< 0.001**
HGB (g/dl)	10 ± 1	10.8 ± 1.3	11.7 ± 1.4	10.8 ± 1.3	10.2 ± 1.2	9.9 ± 1.2	0.6	< 0.001**
RBC (10^6^/µL)	3.5 ± 0.4	3.9 ± 0.5	4.2 ± 0.5	3.8 ± 0.5	3.6 ± 0.5	3.5 ± 0.5	0.65	< 0.001**
HCT (%)	31.8 ± 3.2	34.3 ± 3.4	36.8 ± 3.7	33.9 ± 3.2	32.2 ± 3.1	31.1 ± 3	0.68	< 0.001**
Ca (mg/dl)	7.9 ± 0.9	8.6 ± 7	9.1 ± 0.6	8.6 ± 0.8	8.6 ± 0.7	8.3 ± 0.7	0.3	< 0.001**
P (meq/lit)	5.6 ± 1.5	5 ± 1.2	4.3 ± 0.6	5.4 ± 1.4	5.9 ± 1	5.3 ± 1	0.3	< 0.001**
PTH (pg/ml)	441 (264—567)	290 (224—391)	245 **†** (173—286)	270 (180—418)	342 (245—476)	312 (243—407)	0.8	NA
GNRI	92.8 ± 4.6	96.1 ± 5.1	101.1 ± 4.1	96 ± 5.5	94.5 ± 4.9	92.7 ± 5	0.5	< 0.001**
6 MWT (m)	309 ± 86	348 ± 92	381 ± 99	323 ± 78	304 ± 83	283 ± 87	0.9	< 0.001**

*Alb* Albumin, *HGB* Hemoglobin, *RBC* Red blood cells, *HCT* Hematocrit, *Ca* serum calcium, *P* serum phosphorous,

*PTH* Parathyroid hormone, *GNRI* Geriatric Nutritional Risk Index, *6 MWT* 6-min walk test

Values are as mean ± standard deviation or median (interquartile)

Group × Time is an interaction from a two-way repeated-measures analysis of the effect of time from baseline to 6 months

^*^p < 0.05 significant

^**^p < 0.01 highly significant

^†^*p* < 0.01 highly significant within-group difference, analyzed by Friedman test

### Safety

No treatment-related serious adverse effects were observed during the period of the study. During the intervention period only one of the patients had muscle cramp after two sessions of the exercise and one of the patients had bleeding from fistula when she was doing the exercises. Of course, it was not serious or harmful.

## Discussion

This study showed that intradialytic exercise for 6 months improved subsequent survival in adult patients receiving HD for 12 months. Furthermore, compared with controls, intradialytic exercise caused potentially beneficial improvements in selected laboratory parameters (serum albumin, hemoglobin, red blood cell count, serum calcium, serum PTH, serum phosphorus), physical function (6MWT) and nutritional status (GNRI) during the 6-month intervention period.

The finding of a survival benefit from intradialytic exercise is in keeping that of with previous observational studies investigating the association between increased physical activity and survival in patients receiving dialysis [10, 22]. In a prospective, multinational study of 6147 patients receiving HD in centers across Argentina and Europe, Bernier-Jean et al. [10] found that, compared with self-reported physical inactivity, occasional activity (≤ 1x/week) and frequent activity (> 1x/week) were dose-dependently associated with lower all-cause mortality and cardiovascular mortality, but not non-cardiovascular mortality. This study was potentially limited by social desirability bias, misclassification bias and residual confounding from unmeasured determinants of better health. Similarly, in a systematic review of 11 observational studies involving patients with kidney failure (6 in HD patients, 3 in kidney transplant recipients, 2 in both HD and PD patients), Martins et al. [22] demonstrated that physical activity (self-reported in 10 studies) was generally inversely associated with mortality. However, high degrees of statistical, clinical and methodological heterogeneity precluded meta-analysis. This fact, together with the observational designs of the included studies meant that the evidence was very low certainty. Recently, Mallalamaci et al. [23] reported long-term (36 month) follow-up outcomes of the EXCITE study, a prospective, randomized controlled trial of a 6-month home-based walking exercise program in 227 patients on dialysis (192 on HD) at 13 Italian centers. Compared with controls, patients allocated to the exercise arm had a borderline significantly lower risk of the composite outcome of hospitalization or death (HR 0.71, 95% CI 0.50 – 1.00). As this was a secondary outcome of the trial, the findings were hypothesis-generating only. Until the present study, the effect of intradialytic exercise on patient survival had not been evaluated by randomized controlled trial.

The mechanisms underpinning the improved survival of patients performing intradialytic exercise for 6 months may be explained by improvements in a number of factors associated with physical exercise including bone mineral metabolism [24–27], anemia [28], cholesterol [29], endothelial function (by raising the laminar sheer stress bioavailability of nitric oxide) [30], reduced arterial stiffness due to nitric oxide-mediated vasodilation [29], improved coronary blood flow reserve [29], reduced blood pressure [29], increased physical function [31], reduced oxidative stress [29], decreased inflammation [32], reduced sympathetic nervous system activity [29], and promotion of antithrombotic actions that favor fibrinolysis over thrombosis [30]. In the present study, significant improvements were observed in bone mineral metabolism, anemia, nutrition, physical function (as measured by the 6MWT) and nutritional status (as measured by GNRI). Future studies are required to determine the extent to which each of these adaptations to exercise can contribute to overall survival. Prospective cohorts studies have shown that many of these health outcomes significantly predict mortality and/or contribute to increased survival [33–42]. However, such studies have not specifically evaluated responses to exercise training.

A major strength of the present study was the fact that all exercises were tailored according to each individual's functional status within a pre-specified structure. There was also a high participation rate, such that included HD patients exhibited considerable diversity with respect to demographic characteristics and associated comorbidities. The study also evaluated a range of biochemical and functional parameters. The intervention was designed so that it was broadly implementable in most HD centers, including in low resource settings.

Balanced against these strengths, the study also had a number of limitations. The duration of the intradialytic exercise intervention was short (6 months), as was the subsequent follow-up (12 months), meaning that the long-term effects of exercise on patient survival remain uncertain. The small sample size led to an imprecise estimate of the effect of exercise on patient survival. Survival was timed from the end of the intervention, which introduced immortal time bias. Secondary outcome measures exploring the effects of intradialytic exercise and patient survival were only examined during the first 6 months of the study.

## Conclusion

Intradialytic exercise performed for at least 60 min during thrice weekly dialysis sessions improves survival in adult patients receiving HD. Further large-scale studies are warranted.

## Supplementary Information

**Additional file 1:** **Table S1.** Classificationby health markers. **Figure S1.** Intervention design. **Table S2.** Intervention designfor resistance training.

## Data Availability

The data used and/or analyzed during the current study (without any identifying information) are available in Figshare at https://figshare.com and can be accessed with https://doi.org/10.6084/m9.figshare.21075859. The study Protocol also is available in Figshare at https://figshare.com and can be accessed with https://doi.org/10.6084/m9.figshare.20775553.

## Abbreviations

CKD: Chronic Kidney Disease
KRT: kidney replacement therapy
HD: hemodialysis
PD: peritoneal dialysis
CCI: charlson comorbidity index
GNRI: Geriatric Nutritional Risk Index
6MWT: 6-minute walk test
Alb: albumin
HGB: hemoglobin
RBC: red blood cells
Ca: serum calcium
P: serum phosphorous
PTH: parathyroid hormone
GREX: Global Renal Exercise

## References

[1] Ortiz A, Covic A, Fliser D, Fouque D, Goldsmith D, Kanbay M (2014). Board of the EURECA-m Working Group of ERA-EDTA. Epidemiology, contributors to, and clinical trials of mortality risk in chronic kidney failure. Lancet..

[2] United States Renal Data System (2018). Epidemiology of kidney disease in the United States.

[3] Deligiannis A, D'Alessandro C, Cupisti A (2021). Exercise training in dialysis patients: impact on cardiovascular and skeletal muscle health. Clin Kidney J..

[4] Figliuzzi M, Remuzzi G, Remuzzi A (2014). Renal bioengineering with scaffolds generated from rat and pig kidneys. Nephron Exp Nephrol.

[5] Kurella Tamura M, Covinsky KE (2009). Functional status of elderly adults before and after initiation of dialysis. N Engl J Med.

[6] SONG Initiative. The SONG Handbook. Version 1.0. 2017, Sydney. Available at songinitiative.org/reports-and-publications/.

[7] Manera KE, Tong A, Craig JC, Shen J, Jesudason S, Cho Y (2019). An international Delphi survey helped develop consensus-based core outcome domains for trials in peritoneal dialysis. Kidney Int.

[8] Artom M, Moss-Morris R, Caskey F, Chilcot J (2014). Fatigue in advanced kidney disease. Kidney Int.

[9] O'Hare AM, Tawney K, Bacchetti P, Johansen KL (2003). Decreased survival among sedentary patients undergoing dialysis: results from the dialysis morbidity and mortality study wave 2. Am J Kidney Dis.

[10] Bernier-Jean A, Wong G, Saglimbene V, Ruospo M, Palmer SC, Natale P (2021). Self-reported physical activity and survival in adults treated with hemodialysis: a DIET-HD cohort study. Kidney Int Rep.

[11] Huang M, Lv A, Wang J, Xu N, Ma G, Zhai Z (2019). Exercise training and outcomes in hemodialysis patients: systematic review and meta-analysis. Am J Nephrol.

[12] Parker K (2016). Intradialytic Exercise is Medicine for Hemodialysis Patients. Curr Sports Med Rep..

[13] Lu Y, Wang Y, Lu Q (2019). Effects of exercise on muscle fitness in dialysis patients: a systematic review and meta-analysis. Am J Nephrol.

[14] Liu J, Huang Z, Gilbertson DT, Foley RN, Collins AJ (2010). An improved comorbidity index for outcome analyses among dialysis patients. Kidney Int.

[15] ATS Committee on Proficiency Standards for Clinical Pulmonary Function Laboratories (2002). ATS statement: guidelines for the six-minute walk test.. Am J Respir Crit Care Med.

[16] Bouillanne O, Morineau G, Dupont C, Coulombel I, Vincent JP, Nicolis I (2005). Geriatric Nutritional Risk Index: a new index for evaluating at-risk elderly medical patients. Am J Clin Nutr.

[17] Yamada K, Furuya R, Takita T, Maruyama Y, Yamaguchi Y, Ohkawa S (2008). Simplified nutritional screening tools for patients on maintenance hemodialysis. Am J Clin Nutr.

[18] Yoshida M, Nakashima A, Doi S, Maeda K, Ishiuchi N, Naito T (2021). Lower Geriatric Nutritional Risk Index (GNRI) is associated with higher risk of fractures in patients undergoing hemodialysis. Nutrients.

[19] Yajima T, Yajima K, Takahashi H (2021). Association of the erythropoiesis-stimulating agent resistance index and the geriatric nutritional risk index with cardiovascular mortality in maintenance hemodialysis patients. PLoS One.

[20] Xiong J, Wang M, Zhang Y, Nie L, He T, Wang Y (2018). Association of Geriatric nutritional risk index with mortality in hemodialysis patients: a meta-analysis of cohort studies. Kidney Blood Press Res.

[21] Shimoda T, Matsuzawa R, Yoneki K, Harada M, Watanabe T, Matsumoto M, Yoshida A, Takeuchi Y, Matsunaga A (2017). Changes in physical activity and risk of all-cause mortality in patients on maintence hemodialysis: a retrospective cohortstudy. BMC Nephrol.

[22] Martins P, Marques EA, Leal DV, Ferreira A, Wilund KR, Viana JL (2021). Association between physical activity and mortality in end-stage kidney disease: a systematic review of observational studies. BMC Nephrol.

[23] Mallamaci F, D'Arrigo G, Tripepi G, Lamberti N, Torino C, Manfredini F, et al. Long-term effect of physical exercise on the risk for hospitalization and death in dialysis patients: a post-trial long-term observational study. Clin J Am Soc Nephrol. 2022:CJN.03160322. 10.2215/CJN.03160322. 10.2215/CJN.03160322 PMC9435990 35878932

[24] Falahi M.J, Shahidi SH, Farajzadegan Z (2008). The effect of intradialytic exercise on dialysis efficacy, serum phosphate, hemoglobin and blood pressure control and comparison between two exercise programs in hemodialysis patients. J Isfahan Med School.

[25] Salhab N, Alrukhaimi M, Kooman J, Fiaccadori E, Aljubori H, Rizk R, Karavetian M (2019). Effect of Intradialytic exercise on hyperphosphatemia and malnutrition. Nutrients.

[26] Elshinnawy HA, Mohamed AM, Farrag DAB, AbdElgawad MA (2021). Effect of intradialytic exercise on bone profile inhemodialysis patients. Egypt Rheumatol Rehabil.

[27] Makhlough A, Ilali E, Mohseni R, Shahmohammadi S (2012). Effect of intradialytic aerobic exercise on serum electrolytes levels in hemodialysis patients. Iran. J. Kidney Dis.

[28] Reboredo Mde M, Henrique DM, Faria Rde S, Chaoubah A, Bastos MG, de Paula RB (2010). Exercise training during hemodialysis reduces blood pressure and increases physical functioning and quality of life. Artif Organs.

[29] Bronas UG (2009). Exercise training and reduction of cardiovascular disease risk factors in patients with chronic kidney disease. Adv Chronic Kidney Dis.

[30] Van Craenenbroeck AH, Van Craenenbroeck EM, Kouidi E, Vrints CJ, Couttenye MM, Conraads VM (2014). Vascular effects of exercise training in CKD: current evidence and pathophysiological mechanisms. Clin J Am Soc Nephrol.

[31] Clarkson MJ, Bennett PN, Fraser SF (2019). Exercise interventions for improving objective physical function in patients with end-stage kidney disease on dialysis: a systematic review and meta-analysis. Am J Physiol Renal Physiol.

[32] Viana JL, Kosmadakis GC, Watson EL, Bevington A, Feehally J, Bishop NC (2014). Evidence for anti-inflammatory effects of exercise in CKD. J Am SocNephrol.

[33] Tentori F, Blayney MJ, Albert JM, Gillespie BW, Kerr PG, Bommer J (2008). Mortality risk for dialysis patients with different levels of serum calcium, phosphorus, and PTH: The Dialysis Outcomes and Practice Patterns Study (DOPPS). Am J Kidney Dis.

[34] Nakai S, Akiba T, Kazama J, Yokoyama K, Fukagawa M, Tominaga Y (2008). Effects of serum calcium, phosphorous, and intact parathyroid hormone levels on survival in chronic hemodialysis patients in Japan. Ther Apher Dial.

[35] Toft G, Heide-Jørgensen U, van Haalen H, James G, Hedman K, Birn H (2020). Anemia and clinical outcomes in patients with non-dialysis dependent or dialysis dependent severe chronic kidney disease: a Danish population-based study. J Nephrol.

[36] Rosenberger J, Kissova V, Majernikova M, Straussova Z, Boldizsar J (2014). Body composition monitor assessing malnutrition in the hemodialysis population independently predicts mortality. J Ren Nutr.

[37] Kopple JD (1997). Nutritional status as a predictor of morbidity in maintenance dialysis patients. ASAIO J..

[38] Lacquaniti A, Bolignano D, Campo S, Perrone C, Donato V, Fazio MR (2009). Malnutrition in the elderly patient on dialysis. Ren Fail.

[39] Panichi V, Cupisti A, Rosati A, Di Giorgio A, Scatena A, Menconi O, Bozzoli L, Bottai A (2014). Geriatric nutritional risk index is a strong predictor of mortality in hemodialysis patients: data from the Riscavid cohort. J Nephrol.

[40] Sietsema KE, Amato A, Adler SG, Brass EP (2004). Exercise capacity as a predictor of survival among ambulatory patients with end-stage renal disease. Kidney Int.

[41] Morishita S, Tsubaki A, Shirai N (2017). Physical function was related to mortality in patients with chronic kidney disease and dialysis. Hemodial Int.

[42] Kohl LM, Signori LU, Ribeiro RA, Silva AM, Moreira PR, Dipp T (2012). Prognostic value of the six-minute walk test in end stage renal disease life expectancy: a prospective cohort study. Clinics (Sao Paulo).

